# Identification and validation of ubiquitination-related signature and subgroups in immune microenvironment of tuberculosis

**DOI:** 10.18632/aging.205198

**Published:** 2023-11-09

**Authors:** Peipei Zhou, Jie Shen, Xiao Ge, Haien Cheng, Yanli Sun, Meng Li, Heng Li, Zhengjun Yi, Zhenpeng Li

**Affiliations:** 1 School of Medical Laboratory, Weifang Medical University, Weifang, Shandong 261053, People’s Republic of China; 2 Engineering Research Institute of Precision Medicine Innovation and Transformation of Infections Diseases, Weifang Medical University, Weifang, Shandong 261053,

**Keywords:** tuberculosis, ubiquitination, biomarker, immune cell infiltration, machine learning

## Abstract

Background: *Mycobacterium tuberculosis* (Mtb) is the bacterial pathogen responsible for causing tuberculosis (TB), a severe public health concern that results in numerous deaths worldwide. Ubiquitination (Ub) is an essential physiological process that aids in maintaining homeostasis and contributes to the development of TB. Therefore, the main objective of our study was to investigate the potential role of Ub-related genes in TB.

Methods: Our research entailed utilizing single sample gene set enrichment analysis (ssGSEA) in combination with several machine learning techniques to discern the Ub-related signature of TB and identify potential diagnostic markers that distinguish TB from healthy controls (HC).

Results: In summary, we used the ssGSEA algorithm to determine the score of Ub families (E1, E2, E3, DUB, UBD, and ULD). Notably, the score of E1, E3, and UBD were lower in TB patients than in HC individuals, and we identified 96 Ub-related differentially expressed genes (UbDEGs). Employing machine learning algorithms, we identified 11 Ub-related hub genes and defined two distinct Ub-related subclusters. Notably, through GSVA and functional analysis, it was determined that these subclusters were implicated in numerous immune-related processes. We further investigated these Ub-related hub genes in four TB-related diseases and found that TRIM68 exhibited higher correlations with various immune cells in different conditions, indicating that it may play a crucial role in the immune process of these diseases.

Conclusion: The observed enrichment of Ub-related gene expression in TB patients emphasizes the potential involvement of ubiquitination in the progression of TB. These significant findings establish a basis for future investigations to elucidate the molecular mechanisms associated with TB, select suitable diagnostic biomarkers, and design innovative therapeutic interventions for combating this fatal infectious disease.

## INTRODUCTION

Preceding the emergence of COVID-19, tuberculosis (TB) held the leading spot as the primary cause of death from a single infectious agent, outpacing HIV/AIDS and malaria. However, due to the impact of the COVID-19 pandemic on public health services, TB-related mortality has experienced a rise for the first time in over 20 years, impeding progress in the slow yet steady fight against this disease [1, 2]. The incidence, progression, and prognosis of TB are not solely linked to the toxicity and quantity of the *Mycobacterium tuberculosis* (Mtb), but also closely tied to the host’s immune function. Since Mtb is a type of intracellular parasitic bacteria, the body’s primary immune response to TB is cellular immunity. Comprehending the root causes and mechanisms of TB can potentially aid in directing clinical diagnosis and treatment, resulting in better clinical outcomes.

Ubiquitin is a conserved protein consisting of 76 amino acids ubiquitously present in all eukaryotes. It binds covalently to target proteins through an isopeptide bond, with the C-terminal glycine of ubiquitin and the epsilon-amino group of a lysine residue on the substrate both playing a role in the formation of this covalent bond [3]. Eukaryotic organisms employ a three-step thioester cascade process, mediated by E1s, E2s, and E3s, to execute modifications involving ubiquitin and other ubiquitin-like (Ub/UBL) molecules [4]. These modifications can be reversed by deubiquitinating enzymes (DUBs), which eliminate the Ub/UBL from proteins that have undergone modifications [5, 6]. Proteins that possess ubiquitin-binding domains (UBDs), known as ubiquitin-binding domain-containing proteins, play a regulatory role in various biological processes *in vivo* by selectively recognizing monoubiquitin and ubiquitin chains with different linkages and lengths [7]. Furthermore, ubiquitin-like domains (ULDs) are integral elements of numerous protein families [8]. Overall, the complex process of ubiquitin signal formation and recognition mechanisms allows for the performance of diverse cellular and physiological functions.

Bacterial pathogens employ various strategies to inhibit host innate immune responses by altering key host signaling pathways and post-translational modifications, thus facilitating infection and survival [9, 10]. Several intracellular pathogens have developed a variety of sophisticated molecular weapons to evade natural defense mechanisms. Among these mechanisms, cellular ubiquitination (Ub), a naturally occurring process, is becoming recognized as one exploited by these pathogens. Ub reduces the survival rate of Mtb by promoting the fusion of autophagosomes and lysosomes [11]. In conclusion, there is a close relationship between ubiquitination and the pathophysiology of TB, although the specific mechanisms are still unclear.

We analyzed gene expression differences between HC and TB samples using the Gene Expression Omnibus (GEO) database to investigate potential pathogenesis. Differential genes and ubiquitination-related (Ub-related) genes were extracted, and their intersection was used to find the differentially expressed Ub-related genes (UbDEGs). UbDEGs were identified using multiple machine learning algorithms. Based on the 11 hub UbDEGs expression patterns, we grouped 565 TB patients into two subgroups associated with Ub, and then examined the differences in immune cells between the two subgroups. Here, we integrated the expression profiles of TB and HC using array analysis to comprehensively describe the Ub-related transcriptional features of TB and explore potential diagnostic biomarkers that distinguish TB from HC.

## MATERIALS AND METHODS

### Data collection

The GEO database (http://www.ncbi.nlm.nih.gov/geo) is a repository for public genome datasets. For this study, gene expression profiles were downloaded from GEO, specifically GSE157657, GSE62525, GSE83456, GSE93272, GSE76925, GSE166253, and GSE31210. The platform contains an explanation document that aims to link the probes with their respective genes.

### Identification of DEGs and gene ontology enrichment analysis

To identify differentially expressed genes (DEGs) between the TB and HC, the R package “limma” was utilized [12]. The criteria of filtering were established as follows: *P* < 0.05 and |log2 fold change| > 0.264 or |log2 fold change | > 0.585. To undertake Gene Ontology (GO) enrichment analysis and Kyoto Encyclopedia of Genes and Genomes (KEGG) analysis for the DEGs, we utilized the DAVID tool (https://david.ncifcrf.gov/). Enriched GO pathways and KEGG pathways were determined based on the *P*-value cut-off criterion of < 0.05.

### ssGSEA algorithm calculates the function score

We retrieved ubiquitination terms from the iuucd database (http://iuucd.biocuckoo.org/) to specifically select the relevant functional items for assessing disparities in ubiquitination-related functions within the samples. Next, we applied the single sample gene set enrichment analysis (ssGSEA) algorithm using the R package “GSVA” to score each piece. A higher score indicates a greater relative gene expression level associated with the specific functional item. Based on this scoring metric, we evaluated the activation state in relation to the particular functional item of interest.

### Machine learning

To improve the regularity, interpretability, and predictive accuracy of predictive models and to select relevant variables for model incorporation, we utilized a Least Absolute Shrinkage and Selection Operator (LASSO) regression approach [13]. Support Vector Machine (SVM) method, a technique that establishes a threshold between categories for prediction based on one or more feature vectors, was implemented for analyzing the data [14]. Random forest (RF) approaches were also employed for predicting continuous variables accurately, precisely without significant fluctuations [15]. eXtreme Gradient Boosting (Xgboost) ensemble learning algorithm was also used, which utilizes decision trees as base learners [16]. These machine learning analyses (LASSO, SVM-RFE, Xgboost and RF) were performed using the R packages “glmnet,” “kernlab,” “randomForest,” and “xgboost,” respectively. The genes identified through the intersection of these analyses were considered to be hub genes related to ubiquitination in the diagnosis of TB.

### Constructing the nomogram model

The carefully selected prediction variables (clinical relevance and utility) were utilized to build and validate the nomogram model [17]. Following the final multivariable logistic regression analysis, the nomogram was created in order to provide a visual instrument for evaluating individual risk projections for constipation in TB patients. The effectiveness of the prediction model was evaluated by calculating the area under the receiver operating characteristic curve. Furthermore, we assessed the clinical practicality of the prediction model by utilizing the decision curve analysis (DCA) method to determine net benefits.

### Subclusters analysis with 11 Ub-related hub genes

The TB samples were subject to an unsupervised hierarchical clustering analysis using the “ConsensusClusterPlus” R package [18] based on the expression of 11 Ub-related genes. Subsequently, we employed GSVA [19] to elucidate the function between the Ub-related subclusters identified through the clustering mentioned above analysis.

### Gene set variation analysis (GSVA)

To perform GSVA analysis, we downloaded files “h.all.v7.5.1.symbols”, “c2.cp.kegg.v7.5.1.symbols” and “c2.cp.reactome.v7.5.1.symbols” from MSigDB.

GSVA R package was used to calculate the activities of two sets for each sample. DEGs between the two Ub-related subclusters were identified using a significance cut-off of | log2 fold change (FC)| > 0.585 and *P* < 0.05. These DEGs were illustrated using a volcano plot. Subsequently, GO and KEGG enrichment analyses were carried out using the DAVID tool to uncover the biological functions of the identified DEGs.

### Evaluation of immune cell infiltration

The CIBERSORT algorithm was utilized by us to establish the linked cellular immune infiltration with the help of standard LM22 gene signature. Marker information for 22 immune cells was obtained for the purpose of computing the immune cell infiltration in each dataset [20]. Spearman correlation analysis was performed to investigate the correlations between immune cells and hub genes, enabling the exploration of the relationship between these hub genes and immune cells [21].

### Quality control and processing of single cell data

We obtained single cell raw sequencing data (10X Genomics) from previous study, which can be accessed under the accession numbers SRR11038989 and SRR11038994 [22]. The number of expressed genes was calculated for each single cell. Following preliminary quality control procedures, we utilized the R package “Seurat” to perform a standardized analysis workflow. Firstly, we standardized the data using “NormalizeData”, and then conducted principal component analysis via “RunPCA”. Finally, we utilized the k-nearest neighbor classification algorithm through “FindNeighbors” and “FindClusters” to classify single-cell samples into distinct clusters. We then scored each cluster based on the normalized expressions of canonical markers, which were listed in Supplementary Table 1 [23].

### Statistical analysis

R software (version 4.2.2) was utilized to conduct all the statistical analysis. Figure panels were pieced together by Adobe Illustrator (CC 2019). Statistical significance was ascribed to any difference with a *P*-value or adjusted *P*-value falling below 0.05. Spearman’s correlation coefficient was employed to determine the association between continuous variables. Additionally, a *t*-test or Wilcoxon rank-sum test was performed for statistical comparison between two groups. ^*^p < 0.05, ^**^p < 0.01, ^***^p < 0.001.

### Data availability statement

The original contributions presented in the study are included in the article Supplementary Material. Further inquiries can be directed to the corresponding author.

## RESULTS

### The workflow of this study

**Figure 1:**
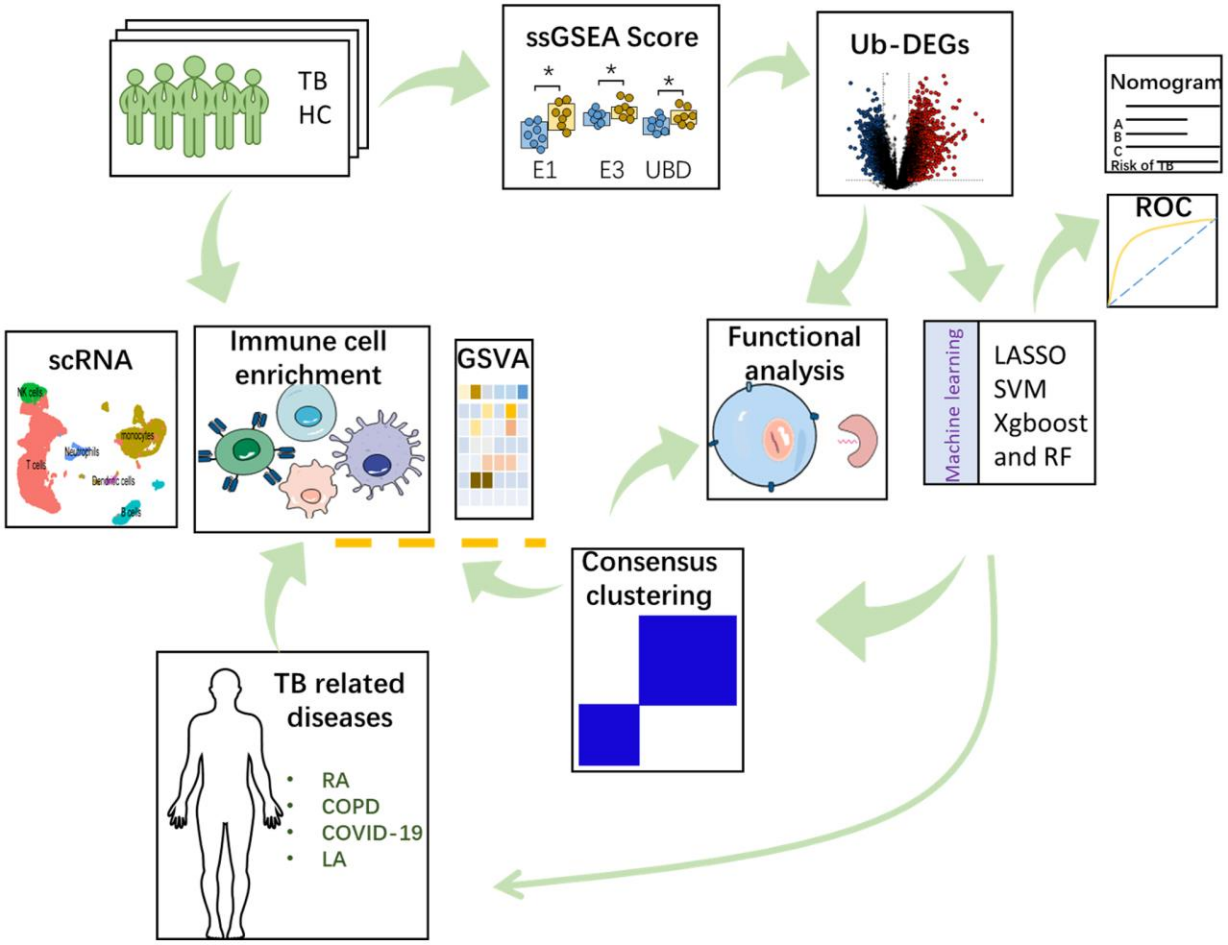
The detailed work process of this study.

The objective of this study was to identify ubiquitination-related biomarkers for TB. Data analysis was conducted using ssGSEA, and machine learning to determine the hub genes that significantly influence the TB response. Subsequently, validation and diagnostic value analyses were performed to assess the potential of hub genes. These findings offer novel perspectives for diagnosing and treating of TB, adding to the existing scientific knowledge in this field. In this work, all mRNA expression-related analyses were achieved by using the gene expression data from the GEO database. The workflow of this study was provided in Figure 1.

### Ub-related DEGs identified in TB

**Figure 2:**
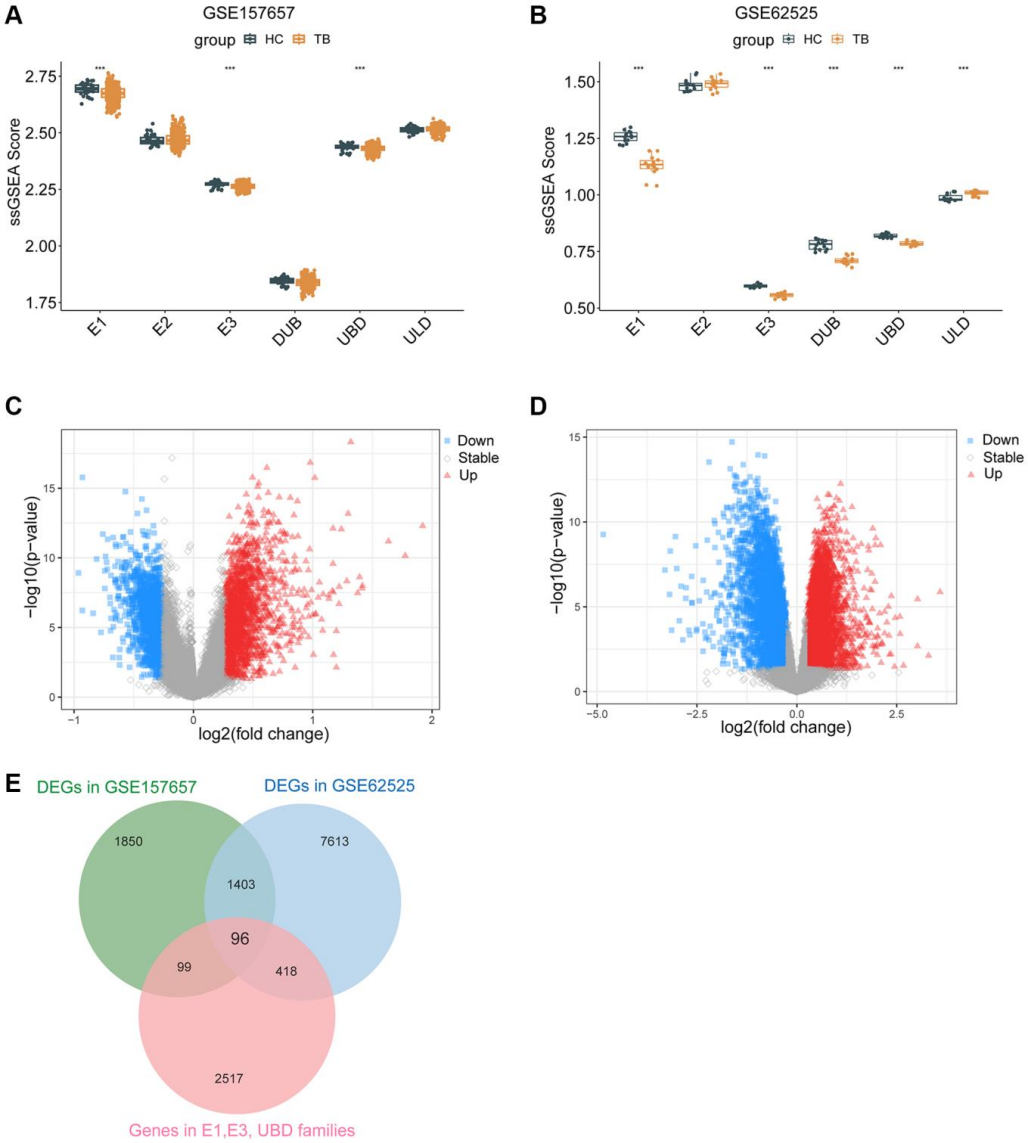
**Ub-related DEGs identified in TB.** ssGSEA score of E1, E2, E3, DUB, UBD, ULD in GSE157657 (**A**) and GSE62525 (**B**) dataset. The volcano plotting of DEGs in GSE157657 (**C**) and GSE62525 (**D**) dataset. (**E**) The overlapping of genes between DEGs and Ub-related genes. DEGs, differentially expressed genes. ssGSEA, single sample gene set enrichment analysis. ^***^p < 0.001.

In total, based on the relevant functional items acquired from the IUUCD database, ssGSEA was conducted to investigate 6 Ub families (E1, E2, E3, DUB, UBD, ULD) in our study and the gene sets in each family were presented in Supplementary Table 2. Among these Ub families, the ssGSEA score of E1, E3, and UBD showed a significant difference in TB and HC in both GSE157657 (Figure 2A) and GSE62525 dataset (Figure 2B). Compared to HC, the abundance of Ub-related genes (E1, E3, and UBD) significantly decreased in TB.

Next, we analyzed GSE157657 and GSE62525 datasets and identified 3448 and 9530 DEGs (|log2 FC| > 0.264 and *P* < 0.05), respectively (Figure 2C, 2D). Of these, 1874 and 5049 genes were upregulated, while 4481 and 1574 genes were downregulated, respectively. To gain a better understanding of TB pathogenesis, we performed a cross-comparison of gene expression profiles. Ultimately, 96 Ub-related DEGs (UbDEGs) were identified as candidate genes for further analysis (Figure 2E).

### Functional and pathway enrichment analysis of UbDEGs

**Figure 3:**
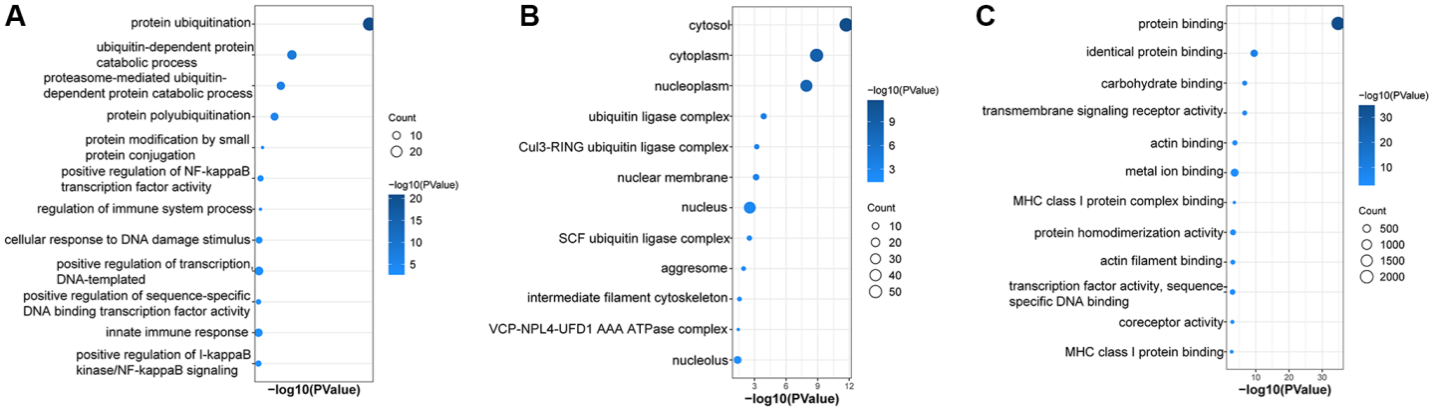
**Enrichment in GO analyses based on UbDEGs.** Enrichment in GO BP (**A**), CC (**B**) and MF (**C**) analysis. Abbreviations: GO: Gene Ontology; BP: Biological process; CC: Cellular component; MF: Molecular function.

GO and KEGG enrichment pathway analysis were performed on the 96 UbDEGs to investigate their potential roles. As expected, the GO analysis indicated significant enrichment of ubiquitination-related biological processes (such as protein ubiquitination, proteasome-mediated ubiquitin-dependent protein catabolic process, ubiquitin-dependent protein catabolic process). The UbDEGs were also enriched in immune-related biology processes (for example, regulation of immune system process, innate immune response) (Figure 3A). The KEGG analysis identified significant enrichment of the Ubiquitin-mediated proteolysis and Shigellosis signaling pathway among the DEGs. All these results further indicated that these UbDEGs were also closely related to immune function. Additionally, we observed enrichment in cellular compartments such as cytosol, cytoplasm, nucleoplasm, and ubiquitin ligase complex (Figure 3B). Furthermore, in terms of molecular function, identical protein binding, protein binding, carbohydrate binding and transmembrane signaling receptor activity were found to be enriched (Figure 3C).

### Identification of the Ub-related hub genes via machine learning

**Figure 4:**
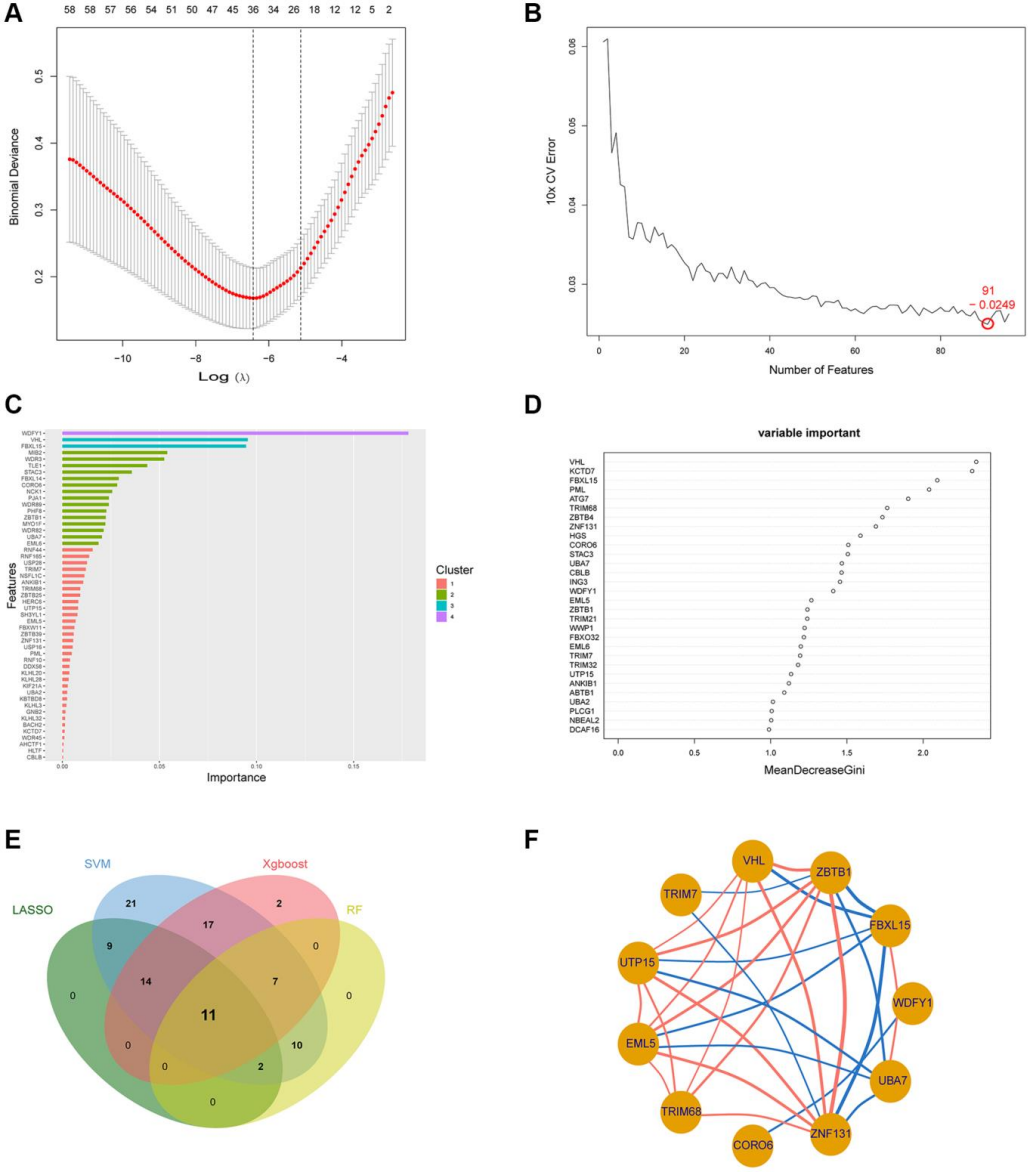
**Employment of machine learning techniques in identifying the Ub-related hub gene.** (**A**–**D**) Construction of Ub-related genes using LASSO, SVM, Xgboost, and RF. (**E**) The overlapping of hub genes between the four machine learning algorithms illustrated by Venn diagram. (**F**) The relationship between the 11 Ub-related hub genes. Abbreviations: LASSO: least absolute shrinkage and selection operator; SVM: support vector machine; XGBoost: eXtreme Gradient Boosting; RF: random forest.

96 UbDEGs as candidate genes were applied for machine learning. We applied the LASSO and selected λ.min to identify 26 Ub-related genes as significant genes (Figure 4A). Then, through SVM with 10-fold cross-validation, 91 Ub-related genes were obtained (Figure 4B). Using Xgboost, and RF algorithms, we ranked the genes based on the importance and extracted the top 51 and top 30 (Figure 4C, 4D), respectively. Ultimately, we identified 11 Ub-related hub genes, which included WDFY1, FBXL15, ZBTB1, VHL, TRIM7, UTP15, EML5, TRIM68, CORO6, ZNF131, and UBA7 (Figure 4E). Moreover, most of the Ub-related hub genes were highly correlated with one another (Figure 4F).

**Figure 5:**
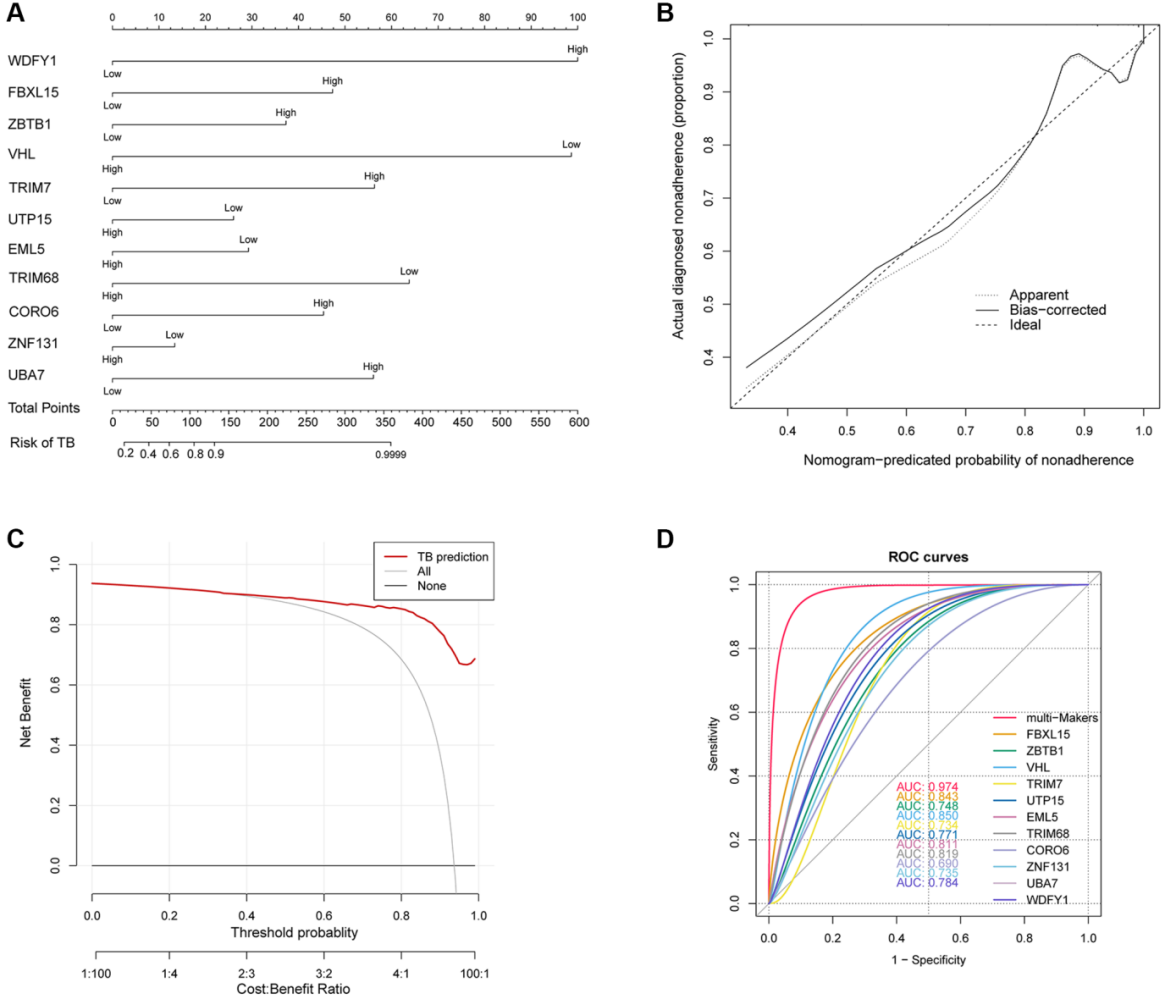
(**A**) Developed nonadherence nomogram. (**B**) The calibration curves for predicting nonadherence using the nomogram in the cohort are illustrated. (**C**) Decision curve analysis for the nomogram. (**D**) ROC curve of Ub-related hub genes in TB diagnosis.

To predict the risk of TB, a nomogram prediction model was constructed (Figure 5A). We evaluated the nomogram’s performance using a calibration curve for TB risk prediction, which showed satisfactory agreement in this cohort (Figure 5B). The C-index of the nomogram was 0.962 (95% CI: 0.94338–0.98062), indicating good discrimination by the model. The nomogram demonstrated sound predictive capability based on its apparent performance. The decision curve analysis for the nonadherence nomogram was depicted in Figure 5C, which shows that the threshold probability range of 0.14-1.00 is clinically significant. Additionally, we analyzed the ROC curves of seven gene signatures and noted that the 11 Ub-related hub genes had the highest AUC value of 0.974 (Figure 5D), indicating their excellent diagnostic value. Similar results have also been observed in GSE62525 and GSE83456 (Supplementary Figure 1A, 1B). These findings revealed that all 11 hub genes exhibit outstanding diagnostic accuracy.

### Evaluation of immune cell infiltration

**Figure 6:**
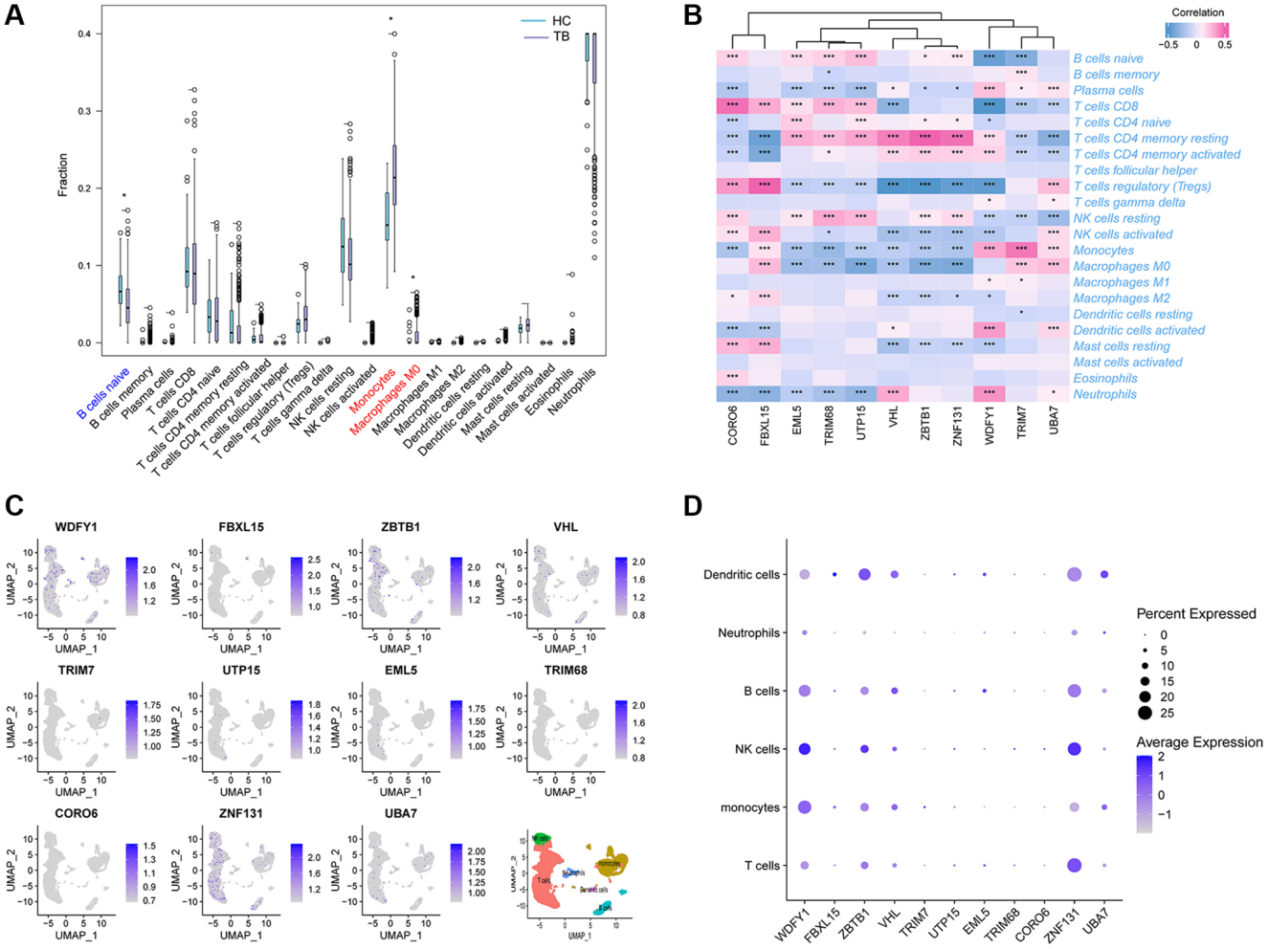
**Immune cell enrichment in patients with TB.** (**A**) The proportion of all 22 types of immune cells. (**B**) The relationship between 11Ub-related hub genes and 22 immune cells. (**C**) The location of the 11 Ub-related genes. (**D**) Dot plot showing the 11 Ub-related genes in each cell cluster. ^*^p < 0.05, ^***^p < 0.001.

Given that UbDEGs were associated with immune-related biological processes (Figure 3A), we investigated immune cell infiltration to gain further insight into the immune regulation of TB. The boxplot (Figure 6A) showed that TB patients exhibited a higher proportion of monocytes and macrophages M0, but a lower proportion of naïve B cells. We then analyzed the correlation between the 11 Ub-related hub genes and immune cells (Figure 6B). It is worth noting that CD4+memory resting T cells and monocytes cells were significantly correlated with all core genes (*P* < 0.001). Our analysis revealed a significant correlation between these genes and immune cells, suggesting their pivotal role in the immune process of TB.

To further verify the correlation between these hub genes and immune cells, we utilized scRNA-seq on PBMCs derived from public datasets to determine the locations of the 11 hub genes. Our findings indicated that WDFY1, ZBTB1, and ZNF131 were dominantly expressed in monocytes, T, NK, B, and dendritic cells, while the expression levels of FBXL15, TRIM7, UTP15, EML5, TRIM68, and CORO6 were relatively low in all subclusters (Figure 6C, 6D).

### Consensus clustering analysis of Ub-related clusters and GSVA of biological pathways

**Figure 7:**
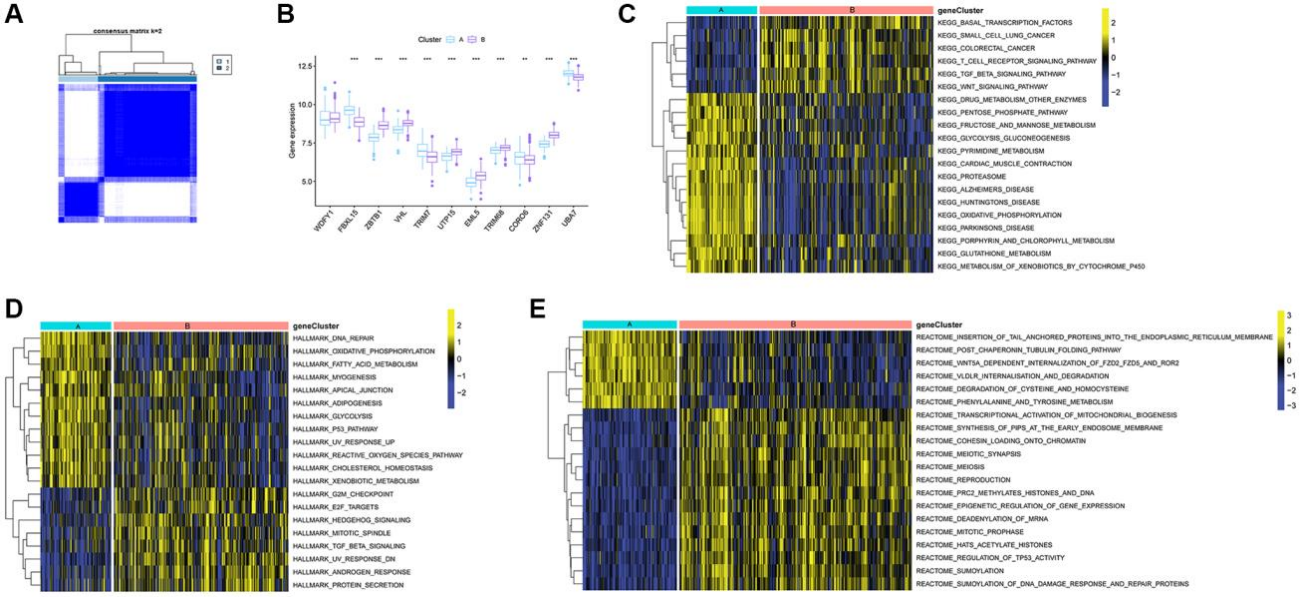
**Identification of Ub-related subclusters in TB samples and GSVA.** (**A**) Subclusters were performed with 11 Ub-related hub genes. (**B**) Boxplot showing the expression level of 11 Ub-related hub genes in cluster A and B. Enrichment of pathways based on the KEGG (**C**), HALLMARK (**D**), and Reatcome pathway (**E**). GSVA, gene set variation analysis. ^**^p < 0.01; ^***^p < 0.001.

Consensus clustering analysis was conducted using the “Consensus Cluster Plus” package in R software. Based on the expression of 11 Ub-related hub genes to investigate more thoroughly the interactions and consistency among DEGs, we established that k = 2 yielded the most consistent grouping (Figure 7A). After that, the 565 TB samples were divided into two distinct subclusters, cluster A (*n* = 163) and cluster B (*n* = 402). The expression levels of 11 hub genes in these two clusters were visualized via a boxplot (Figure 7B). Most Ub-related hub genes showed significantly different expression levels between clusters B and A, except for WDFY1.

By utilizing GSVA analysis, we identified several pathways with differential expression. These pathways were shown by a heatmap (Figures 7C–7E). Compared with cluster B, the expression of KEGG pathways linked with Basal transcription factors, small cell lung cancer, colorectal cancer, T cell receptor signaling pathway, TGF-beta signaling pathway were dramatically reduced in cluster A, while drug metabolism other enzymes, pentose phosphate pathway, and fructose and mannose metabolism were higher in cluster A (Figure 7C). In cluster A, Hallmark activities of DNA repair, oxidative phosphorylation, fatty acid metabolism, myogenesis were higher, while G2M checkpoint, E2F targets, hedgehog signaling were lower (Figure 7D). According to the Reactome-based pathway, GSVA analysis displayed significant enrichment in insertion of tail anchored proteins into the endoplasmic reticulum membrane, post chaperonin tubulin folding pathway, WNT5A dependent internalization of FZD2 FZD5 and ROR2 in cluster A. In contrast, transcriptional activation of mitochondrial biogenesis, synthesis of PIPs at the early endosome membrane, cohesion loading onto chromatin, and meiotic synapsis were enriched in cluster B (Figure 7E).

### Functional distinctions between the two Ub-related subclusters

**Figure 8:**
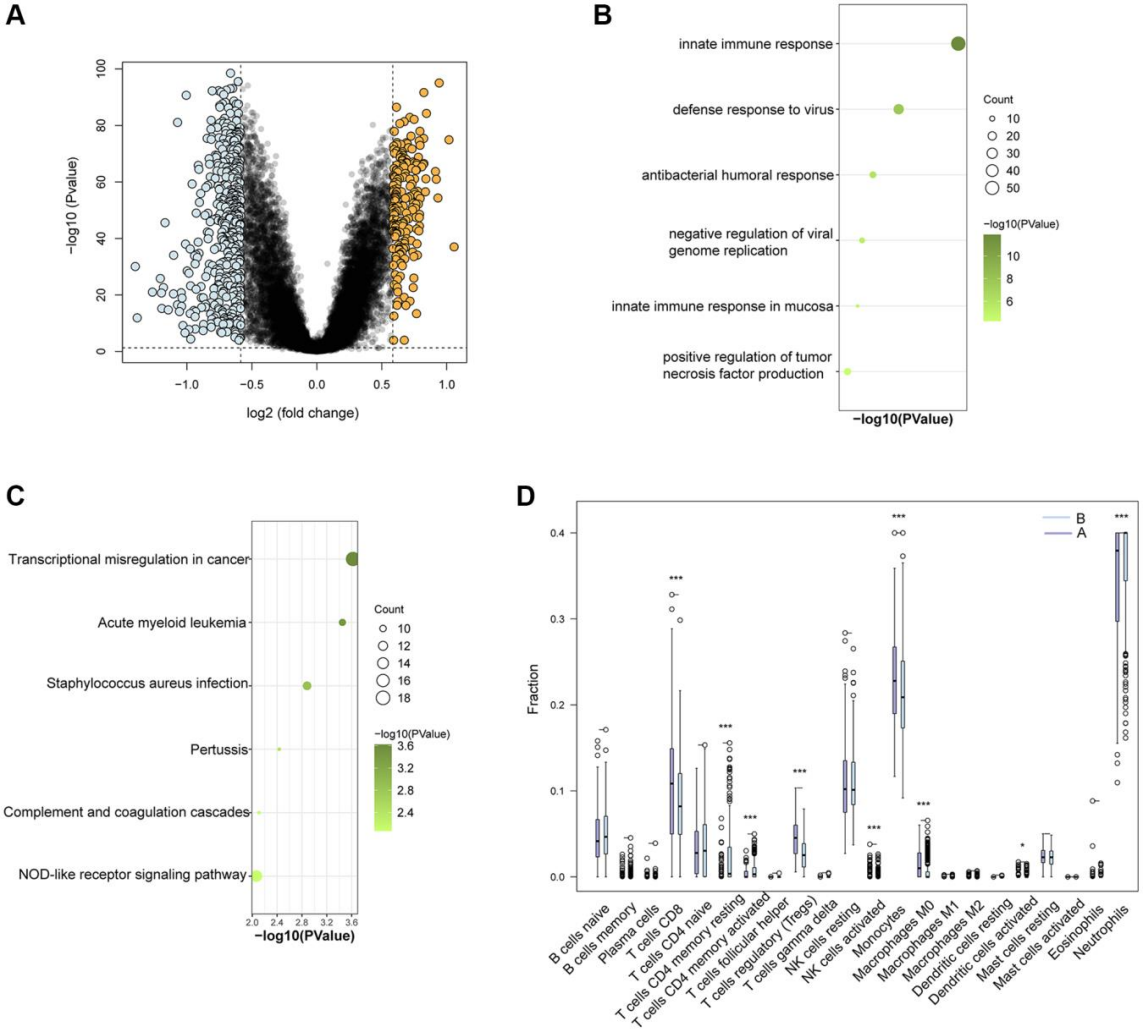
**Functional and immune cell enrichment analysis between Ub-related subcluster.** (**A**) The volcano plotting of DEGs. (**B**) Enrichment items in GO BP and (**C**) KEGG pathway analysis. (**D**) The proportion of all 22 types of immune cells. ^*^p < 0.05; ^***^p < 0.001.

In order to obtain a more profound comprehension of the functional differences between the two subclusters, a differential expression analysis was conducted. A total of 678 DEGs were identified with |log2 fold change| > 0.585 and *P* < 0.05, out of which 210 were upregulated and 468 were downregulated. The volcano plotting was employed to depict the distribution of DEGs between the two subclusters (Figure 8A). Additionally, GO and KEGG enrichment analysis was conducted on the 678 DEGs to better understand their possible molecular processes and functions. The genes showed enrichment in innate immune response, defense response to virus, antibacterial humoral response, negative regulation of viral genome replication, and innate immune response in mucosa, as was indicated by BP- Gene Ontology analysis (Figure 8B). Moreover, the KEGG enrichment analysis illustrated that these genes were predominantly enriched in transcriptional misregulation in cancer, acute myeloid leukemia, *staphylococcus aureus* infection, pertussis, and complement and coagulation cascades (Figure 8C). Immune cell infiltration investigation revealed that the abundance of neutrophils, resting memory CD4 T, activated memory CD4 T, activated NK, and activated Dendritic cells were higher in cluster B. In contrast, CD8 T, regulatory T cells, monocytes, and macrophages M0 were lower in cluster B than cluster A (Figure 8D). All these findings were consistent with GSVA results.

### Ub-related hub genes in TB-related disease

**Figure 9:**
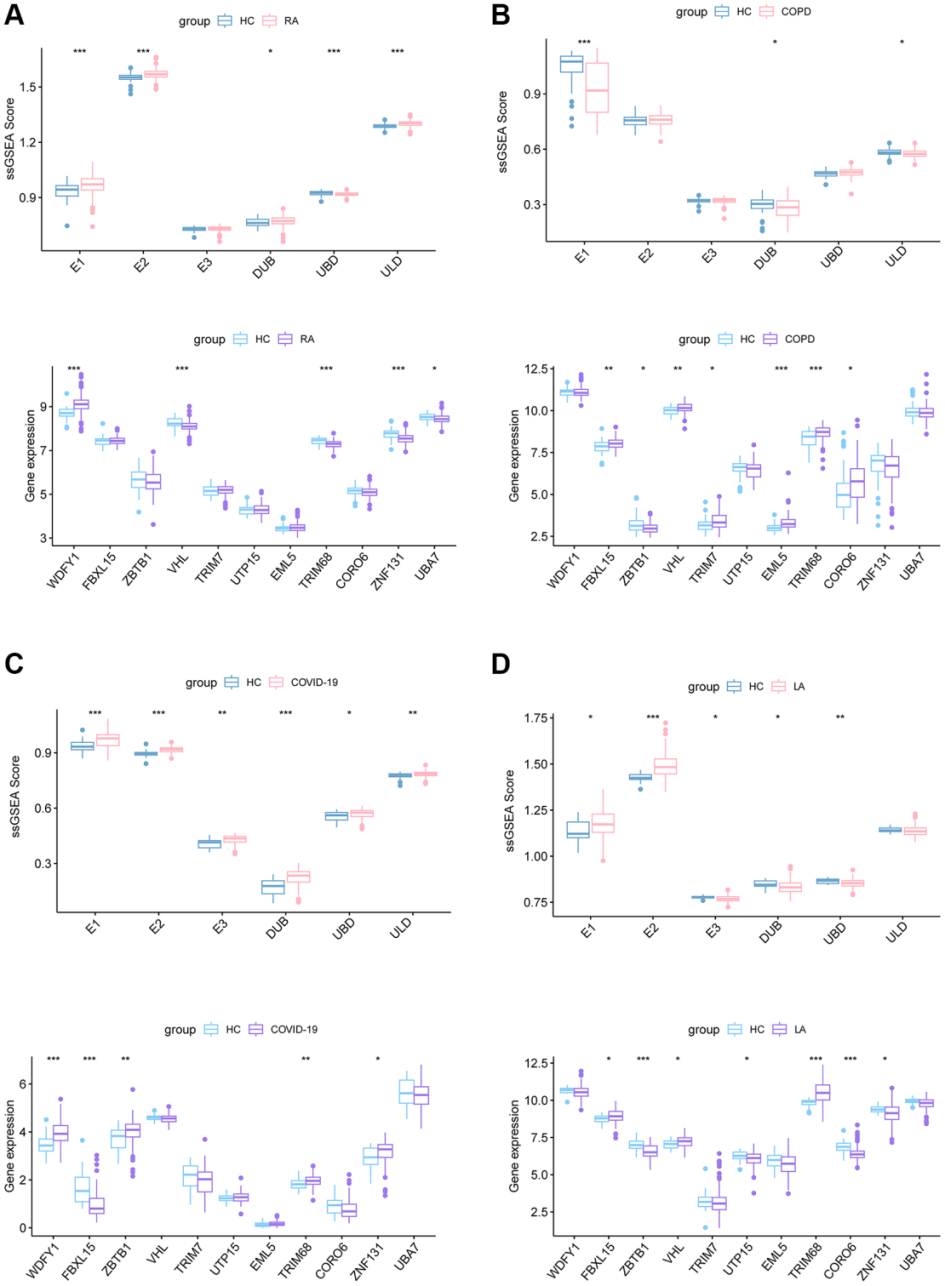
ssGSEA score of E1, E2, E3, DUB, UBD, ULD and the expression of 11 Ub-related hub genes in RA (**A**), COPD (**B**), COVID-19 (**C**), and LA (**D**) dataset. Abbreviations: RA: rheumatoid arthritis; COPD: Chronic obstructive pulmonary disease; COVID-19: corona virus disease 2019; LA: lung adenocarcinomas.

Numerous publications have already reported on the correlation between TB and various diseases, such as rheumatoid arthritis (RA) [24], chronic obstructive pulmonary disease (COPD) [25], corona virus disease 2019 (COVID-19) [26], and lung adenocarcinomas (LA) [27]. To delve further into the potential involvement of Ub-related genes in these diseases, we conducted an analysis using ssGSEA. Compared to HC, the score of E1 family was significantly changed in all TB-related diseases (Figure 9). The E2, DUB and UBD scores showed differently in RA, COVID-19 and LA. The results showed in Figure 9 suggested that ubiquitination may play a part in the development of these conditions. We observed varying levels of gene expression in the samples of these diseases, with TRIM68 standing out as having a significantly different expression across the range of diseases (Figure 9). That may suggest an essential role for TRIM68 in the association between TB and these diseases.

**Figure 10:**
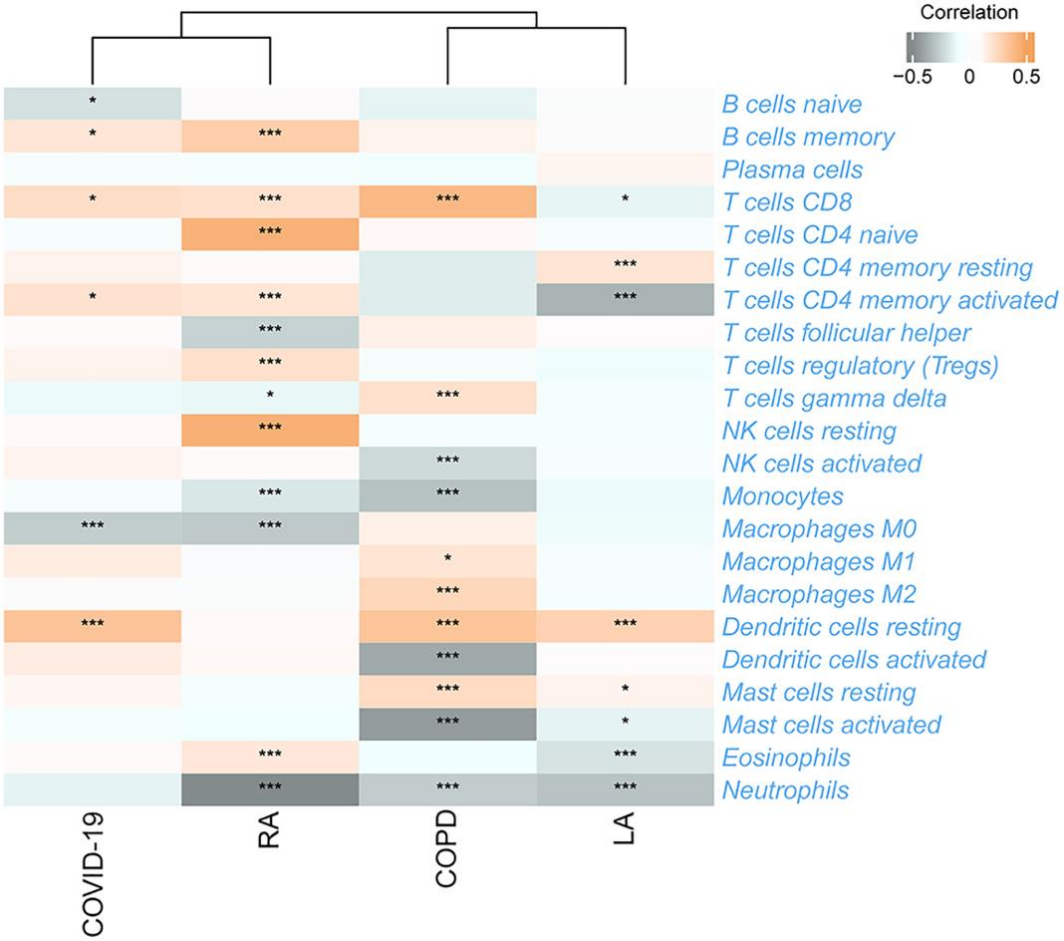
**The relationship between TRIM68 and 22 immune cells in four TB-related diseases.**^ *^p < 0.05; ^***^p < 0.001.

Additionally, TRIM68 displayed stronger correlations with a range of immune cells in various diseases (Figure 10), indicating a possible involvement in the immune processes related to these conditions.

## DISCUSSION

The significant threat that active TB poses to public health spurs us to seek approaches for early detection and treatment to treat TB on a global scale. Owing to the complex immune mechanisms that contribute to the resistance of Mtb infection and the variability in cells involved, obtaining a comprehensive understanding of transcript abundance and their functions in TB pathogenesis. In this context, the results of ssGSEA provide insight into the Ub-related signature of TB and further explore promising diagnostic markers for differentiating TB from HC. E1, E3, and UBD were identified among TB patients as compared to HC. Upon Mtb infection, E1 and E3 facilitate the ubiquitination and degradation of TRAF2 and TAK1, thereby impeding the activation of NF-κB signaling and host innate responses [28]. Host ubiquitin ligases ubiquitinate intracellular Mtb for specific delivery to E3 receptor-mediated phagosomes [11]. Pathogens exploit the host Ubiquitin system in targeting conserved signal cascades, such as the UBD-like motif, to impede innate immunity against Mtb [11].

In total, we identified 96 UbGEGs and conducted GO and KEGG analysis, which indicated that these genes are closely associated with ubiquitination and immune processes. We utilized four machine learning classifiers to select the top 11 hub genes (WDFY1, FBXL15, ZBTB1, VHL, TRIM7, UTP15, EML5, TRIM68, CORO6, ZNF131, and UBA7). To further investigate the relationship between Ub regulators and TB, we analyzed the correlation between hub UbDEGs. The results demonstrated significant evidence of synergistic or antagonistic interactions among Ub-related hub genes in TB samples. Studies have examined the function of WDFY1 protein as a scaffold/recruiting protein for TLR3/4 in the immune system, as well as its involvement in various oncogenic conditions [29]. ZBTB1 plays a role in the regulation of B cell development and differentiation in both peripheral lymphoid organs and bone marrow [30]. Liu et al. discovered that mice with VHL deficiency in T cells exhibit increased vulnerability to Mtb infection due to a decrease in the accumulation of mycobacteria-specific T cells in the lungs with decreased proliferation, altered differentiation, and increased expression of inhibitory receptors [31]. TRIM7 suppresses the replication of enterovirus and facilitates the emergence of a viral variation with escalated pathogenicity [32]. TRIM68, UTP15, CORO6, ZNF131, and UBA7 are associated with tumors [33–36]. Recent literature suggested TRIM7 was the blood signature [37]. The immune cell infiltration results and scRNA analysis revealed that these hub Ub-related genes correlated with the immune cells. Nevertheless, there is less documentation on the significance of these genes in TB, and additional investigations are necessary.

Utilizing consensus clustering analysis based on 11 Ub-related hub genes, we identified two distinct Ub-related subclusters. In terms of subcluster function analyses, we conducted GSVA and found that cluster B showed relatively higher levels of immune-related pathways and lower levels of metabolic-related pathways. GO analysis revealed that DEGs were primarily enriched in immune-related biological processes, while KEGG analysis demonstrated that these genes were predominantly enriched in transcription misregulation in cancer, acute myeloid leukemia, and other pathways. It is well-established that TB can trigger an immune response. CD16- classical monocytes have displayed more significant anti-mycobacterial immune responses during TB infection when compared to CD16+ monocytes. This includes increased migration *in vitro* in response to mycobacterial derivatives, higher production of Reactive Oxygen Species, higher lung migration index, and strong pulmonary infiltration [38]. In contrast, CD16+ monocytes have been linked to promoting bacterial resilience [39]. CD8+ T and NK cells are essential components of the immune response to tuberculosis, with critical roles played by macrophages, effector CD4+ T lymphocytes, and IFN-γ, produced by Th1 cells and triggers macrophage activation [40]. The ssGSEA method was applied to calculate the score of Ub-related signatures, which suggested that Ub-related genes played a role in TB-related diseases. TRIM68 was implicated in the pathogenesis of TB and other TB-related diseases, and was also linked to the immune response underlying these diseases.

## CONCLUSIONS

To sum up, our research thoroughly characterized 11-Ub related hub genes and identified crucial molecular disparities between TB and HC through a combination of array-based expression profiling and scRNA-seq. These findings underscore the importance of longitudinal studies to examine whether biomarkers of recent infection can predict the likelihood of developing TB, and to assist in tracing current transmission in populations. Moreover, the identified Ub-related genes could potentially be targeted for TB treatment.

## Supplementary Materials

Supplementary Figure 1

Supplementary Table 1

Supplementary Table 2
