# Supplementary material for: Identification and validation of ubiquitination-related signature and subgroups in immune microenvironment of tuberculosis
Source: Aging (Albany NY). 2023 Nov 9;15(21):12570–87. doi: 10.18632/aging.205198 (PMC10683621; doi:10.18632/aging.205198)
Supplement: Supplementary Figure 1 [file aging-15-205198-s001.pdf]

SUPPLEMENTARY FIGURE

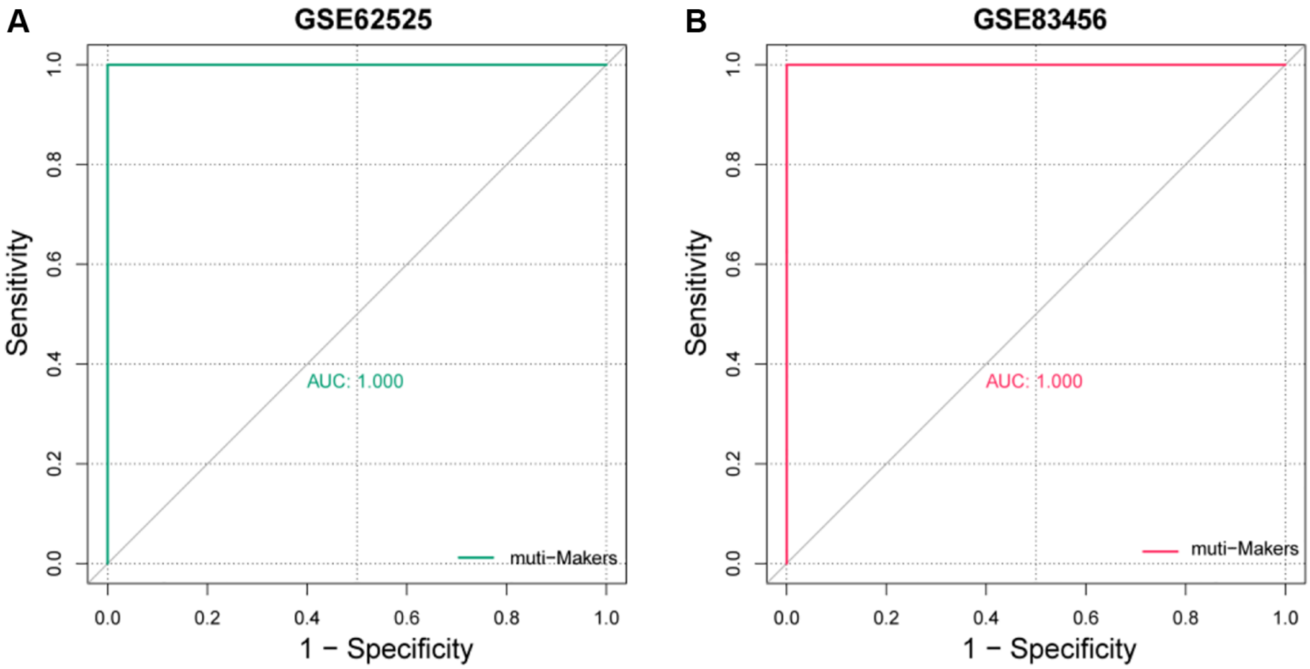

Supplementary Figure 1. ROC curve of Ub-signatures in GSE62525 (**A**) and GSE83456 (**B**) in TB diagnosis.
