# Supplementary material for: Identification and validation of ubiquitination-related signature and subgroups in immune microenvironment of tuberculosis
Source: Aging (Albany NY). 2023 Nov 9;15(21):12570–87. doi: 10.18632/aging.205198 (PMC10683621; doi:10.18632/aging.205198)
Supplement: Supplementary Table 1 [file aging-15-205198-s002.pdf]

SUPPLEMENTARY TABLE

Supplementary Table 1. The markers in different cells type used in this study.

Cells type	Markers
T cells	CD3D, CD3E, TRBC1
NK cells	CD3D, KLRD1, NKG7, KLRC1, FCGR3A
B cells	MS4A1, CD79A, CD79B
Neutrophils	LYZ, CSF3R, CXCR2, FCGR3B
monocytes	LYZ, CD14, FCN1, FCGR3A, S100A9
Dendritic cells	FCER1A, IL3RA, CLEC4C, LILRB4
